# Pasiflora proteins are novel core components of the septate junction

**DOI:** 10.1242/dev.119412

**Published:** 2015-09-01

**Authors:** Myrto Deligiannaki, Abbie L. Casper, Christophe Jung, Ulrike Gaul

**Affiliations:** Gene Center, Department of Biochemistry, Center of Protein Science CIPSM, Ludwigs-Maximilians University, Feodor-Lynen-Str. 25, Munich 81377, Germany

**Keywords:** Septate junction, Blood-brain barrier, Trachea, *Drosophila*, Epithelia

## Abstract

Epithelial sheets play essential roles as selective barriers insulating the body from the environment and establishing distinct chemical compartments within it. In invertebrate epithelia, septate junctions (SJs) consist of large multi-protein complexes that localize at the apicolateral membrane and mediate barrier function. Here, we report the identification of two novel SJ components, Pasiflora1 and Pasiflora2, through a genome-wide glial RNAi screen in *Drosophila*. Pasiflora mutants show permeable blood-brain and tracheal barriers, overelongated tracheal tubes and mislocalization of SJ proteins. Consistent with the observed phenotypes, the genes are co-expressed in embryonic epithelia and glia and are required cell-autonomously to exert their function. Pasiflora1 and Pasiflora2 belong to a previously uncharacterized family of tetraspan membrane proteins conserved across the protostome-deuterostome divide. Both proteins localize at SJs and their apicolateral membrane accumulation depends on other complex components. In fluorescence recovery after photobleaching experiments we demonstrate that pasiflora proteins are core SJ components as they are required for complex formation and exhibit restricted mobility within the membrane of wild-type epithelial cells, but rapid diffusion in cells with disrupted SJs. Taken together, our results show that Pasiflora1 and Pasiflora2 are novel integral components of the SJ and implicate a new family of tetraspan proteins in the function of these ancient and crucial cell junctions.

## Abstract (precis)

**Summary:** Two tetraspan membrane proteins, Pasiflora1 and 2, are essential components of the *Drosophila* septate junction required for epithelial and blood-brain barrier integrity.

## INTRODUCTION

The generation of distinct chemical milieus within the body is essential for metazoan development. This compartmentalization is accomplished by epithelia that impede paracellular diffusion and selectively transport substances via membrane channels and transporters. To provide a barrier, epithelia have a narrow intercellular space, which is sealed by specialized junctions, including tight junctions (TJs) in vertebrates and septate junctions (SJs) in invertebrates (Noirot-Timothée et al., 1978; Tepass and Hartenstein, 1994; Schwabe et al., 2005). SJs are the ancestral sealing junctions and are found in all invertebrates from sponges to arthropods but are also present in vertebrates (Leys and Riesgo, 2012). In electron micrographs, SJs appear as an array of regularly spaced septa, which operate by extending the travel distance for solutes through the paracellular route. SJs are found in both primary epithelia, such as epidermis, trachea and hindgut, and secondary epithelia, which develop through mesenchymal-epithelial transition, such as the blood-brain barrier (BBB) and midgut (Tepass, 1997; T. Schwabe, X. Li and U.G., unpublished). The BBB ensheaths the nervous system and is required to maintain its homeostasis. Owing to the high potassium content of the hemolymph, animals with a defective BBB die of paralysis. In *Drosophila*, the BBB is a squamous epithelium established late in embryogenesis by SJ-forming subperineurial glia (SPG) (Edwards et al., 1993; Schwabe et al., 2005). Here, in addition to providing a paracellular barrier, SJs also serve as a fence for the diffusion of proteins across the lateral membrane (T. Schwabe, X. Li and U.G., unpublished). Molecularly and functionally homologous SJs are found in vertebrates at the node of Ranvier, where they form the paranodal junction between axons and myelinating glia (Poliak and Peles, 2003).

The SJ consists of a large multi-protein complex. In *Drosophila*, more than 20 proteins have been characterized that when missing lead to disruption of SJs and loss of barrier integrity (Izumi and Furuse, 2014). Most of these are transmembrane (TM) and lipid-anchored proteins that localize at the SJ, such as the claudins Sinuous (Sinu) (Wu et al., 2004), Megatrachea (Mega; Pickel – FlyBase) (Behr et al., 2003) and Kune-kune (Kune) (Nelson et al., 2010), the cell adhesion molecules Neurexin IV (Nrx-IV) (Baumgartner et al., 1996), Contactin (Cont) (Faivre-Sarrailh et al., 2004), Neuroglian (Nrg) (Genova and Fehon, 2003), Lachesin (Lac) (Llimargas et al., 2004) and Fasciclin III (FasIII, or Fas3) (Woods et al., 1997), the sodium pump with its two subunits ATPα and Nervana 2 (Nrv2) (Genova and Fehon, 2003; Paul et al., 2003), Melanotransferrin (Transferrin 2 – FlyBase) (Tiklová et al., 2010) and Macroglobulin complement-related (Mcr) (Bätz et al., 2014; Hall et al., 2014). The complex also includes the intracellular scaffold proteins Coracle (Cora) (Fehon et al., 1994) and Varicose (Vari) (Wu et al., 2007) that interact with the cytoplasmic tails of membrane proteins and connect them to the actin cytoskeleton. A hallmark of SJ proteins is that they are interdependent for localization, and removal of one component is sufficient to destabilize the whole complex. In addition, half of the known SJ proteins can be co-immunoprecipitated from tissue extracts and detected by mass spectrometry (MS), further suggesting that they function together in a multi-protein complex (Genova and Fehon, 2003; Faivre-Sarrailh et al., 2004; Tiklová et al., 2010; Jaspers et al., 2012). Fluorescence recovery after photobleaching (FRAP) experiments have been instrumental in classifying most SJ proteins as core components based on their limited mobility after photobleaching and the observation that upon loss of function other SJ proteins diffuse rapidly into the bleached region due to impaired complex formation (Laval et al., 2008; Oshima and Fehon, 2011).

Accompanying epithelial morphogenesis, SJs are remodeled into mature junctions. At embryonic stage 12, SJ proteins accumulate along the lateral membrane of columnar epithelial cells. Subsequently, they gradually localize at more apical compartments and by stage 15 are restricted to the apicolateral membrane, basal to adherens junctions. The Ly-6 proteins Crooked (Crok), Crimpled (Crim) and Coiled (Cold) are required for SJ formation; however, they do not reside at SJs and instead localize to cytoplasmic puncta. In Ly-6 mutants, the FRAP kinetics of SJ proteins mirrors that of core complex mutants and therefore Ly-6 proteins are thought to be involved in the assembly of SJ (sub)complexes in an intracellular compartment (Nilton et al., 2010; Oshima and Fehon, 2011). The subsequent relocalization of SJs requires endocytosis from the basolateral membrane and recycling to the apicolateral compartment (Tiklová et al., 2010; Oshima and Fehon, 2011). Gliotactin (Gli) and Discs-large (Dlg; Dlg1 – FlyBase) localize at SJs (Woods and Bryant, 1991; Woods et al., 1997; Schulte et al., 2003) but, in contrast to core components and Ly-6 proteins, upon their loss of function the complex is properly formed and SJ proteins, although mislocalized, retain their restricted mobility (Oshima and Fehon, 2011). Together with a lack of physical interactions with SJ components, this result suggests that Gli and Dlg are required for complex localization rather than its assembly (Ward et al., 1998; Schulte et al., 2003, 2006).

In contrast to SJs, TJs localize apically of the zonula adherens and in electron microscopy appear as a series of fusions of adjacent membranes. Although the set of proteins that composes the TJ is different from that of the SJ, the two complexes share a key molecular component, the claudins. Claudins are a tetraspan membrane family of 20-34 kDa proteins with intracellular N- and C-termini and constitute a main component of TJs. The larger first extracellular loop contains a claudin family signature motif and bears critical residues that define TJ charge and size selectivity in a tissue-specific manner (Günzel and Yu, 2013). Claudins are part of a large protein clan, comprising the PMP22/EMP/MP20/Claudin (PF00822), MARVEL (PF01284), tetraspanin (PF00335), connexin (PF00029) and innexin (PF00876) families, which share the same overall topology but differ in size and motif composition of extracellular and intracellular domains. Many members of this clan can form homo- and heterotypic oligomers on the same and neighboring membranes and play essential roles in junctional complexes, including TJs, gap junctions and the casparian strip of plants, as well as in membrane traffic and fusion events. Claudins have been shown to interact with other tetraspan proteins such as occludins, tetraspanins and MARVEL, as well as cell adhesion proteins and receptors. Similarly, tetraspanins form microdomains in the plasma membrane, in which cell adhesion proteins, TM receptors and their signaling components are enriched and, thereby, are thought to be modulated in their activity (Sánchez-Pulido et al., 2002; Hua et al., 2003; Hemler, 2005; Cording et al., 2013; Simske, 2014; Van Itallie and Anderson, 2014; Roppolo et al., 2014).

Here we identify and characterize two new core components of the SJ, Pasiflora1 and Pasiflora2, which are part of a novel tetraspan protein family that is conserved across the prostostome-deuterostome divide and is characterized by specific sequence features. Both proteins localize at SJs, show interdependence for localization and restricted mobility with known SJ members and are required for the integrity of epithelial barriers. Our work provides new insight into the composition of the SJ and implicates a second family of tetraspan proteins in the development of these crucial cell junctions.

## RESULTS

### Novel pasiflora genes are required for BBB formation

**Fig. 1.**
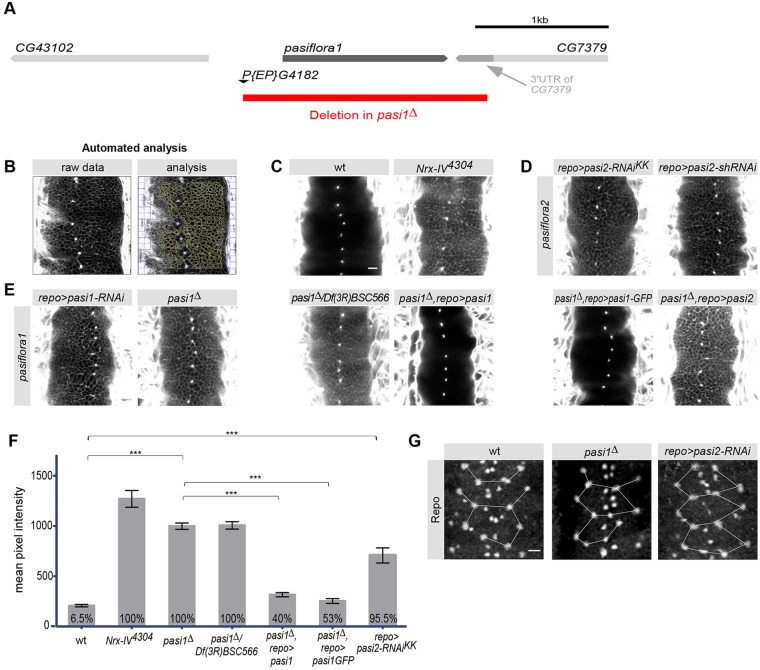
**Pasiflora genes are required for BBB formation.** (A) The *Drosophila* *pasiflora1* genomic region. The deletion spans the whole *pasiflora1* locus and part of the *CG7379* 3′UTR. (B-E) Single confocal sections of 20 h after egg lay (AEL) dye-injected embryos. (B) Example of automated analysis for pixel intensity measurements. The software automatically excludes overexposed areas, such as the body cavity and channels running through the CNS. Dye diffuses into the nerve cord of *Nrx-IV* mutant positive controls (C) and in *pasiflora1* (E) and *pasiflora2* (D) mutants, in contrast to wt embryos (C). Pan-glial overexpression of *pasiflora1* or *pasiflora1-GFP*, but not *pasiflora2*, rescues the phenotype of *pasiflora1^Δ^* (E). Anterior is up. (F) Quantification of the dye penetration assay. Shown is the intensity of dye penetration into nerve cord as measured by mean pixel intensity. The percentage of embryos showing penetration is indicated at the bottom of each column. ***P<0.001, ±s.e.m., *n*=22-139. (G) Ventral surface views of stage 16 embryonic nerve cord stained for Repo. The full complement of SPG is detected in *pasiflora1* and *pasiflora2* mutants. The positions of nuclei are similar between the genotypes, as visualized by overlay of connecting lines. Maximum projections of 4 µm *z*-stacks. Three abdominal neuromeres are shown. Anterior is up. *n*=8-15. Scale bars: 10 µm.

To identify novel genes required for BBB formation, we followed an *in vivo* RNAi approach using 10,450 *UAS-RNAi* strains (75% genome coverage) from the Vienna *Drosophila* Resource Center (VDRC) KK library (Dietzl et al., 2007). To efficiently phenocopy the impaired genotype, *UAS-dicer2* was co-expressed in all screening steps. We initially tested the lines for adult lethality using the strong pan-glial driver *repo-Gal4*. The lines causing lethality or subviability were retested for impaired viability with the SPG-specific but weaker *moody-Gal4*. To directly examine whether the BBB is compromised in the knockdown of the genes, we performed the embryonic dye penetration assay in a selection of candidates (using *repo-Gal4*); in wild-type (wt), the injected dye is excluded from the CNS, but in BBB mutants, such as in *Nrx-IV* embryos, it rapidly diffuses into the nervous system (Fig. 1C,F). To quickly quantify dye accumulation in a systematic fashion, we developed an automated analysis script using Definiens (Fig. 1B).

Among the candidates identified, the lines *CG7713^102223^* and *CG8121^105806^* caused complete adult lethality with *repo-Gal4* and adult subviability with *moody-Gal4* (23% and 17% survivors for 102223 and 105806, respectively; 51% for negative controls). Pan-glial knockdown of both genes resulted in leaky BBB (Fig. 1D-F) and late embryonic lethality (1% hatch; wt, 99%). Interestingly, *CG7713* and *CG8121* belong to one family (With et al., 2003). Inspired by the paralysis resulting from the BBB defect, we named the genes *pasiflora1* (*pasi1*, *CG7713*) and *pasiflora2* (*pasi2*, *CG8121*) from the Greek mythological goddess who induced paralysis in her victims. Our results suggest that the family members *pasiflora1* and *pasiflora2* are novel genes required for BBB formation.

To analyze the phenotypes in complete loss of function, we sought to generate genomic mutants. The viable line *P{EP}G4182* carries a P-element insertion 219 bp upstream of the *pasiflora1* 5′UTR. We created imprecise excisions and isolated a line, *pasiflora1^Δ^*, that deletes the entire *pasiflora1* locus and 59 bp of the *CG7379* 3′UTR (Fig. 1A). *pasiflora1^Δ^* die as late embryos (0% hatch) and have a permeable BBB. A similarly leaky BBB is observed in embryos transheterozygous for *pasiflora1^Δ^* and the deficiency chromosomes *Df(3R)BSC566* and *Df(3R)ED5785*, which uncover the locus. The dye leakage is severe, but weaker than that of the amorphic *Nrx-IV^4304^* SJ mutant (Fig. 1E,F; data not shown). However, *Nrx-IV* is only zygotically expressed (Baumgartner et al., 1996), whereas *pasiflora1* is also maternally provided [see Fig. 3A; BDGP website (http://insitu.fruitfly.org)] (Graveley et al., 2011). To ultimately prove that the glial loss of *pasiflora1* is causing the leaky BBB, we sought to rescue the dye penetration of *pasiflora1^Δ^*. Pan-glial expression of *pasiflora1* restores BBB function (Fig. 1E,F), demonstrating that neither the neighboring *CG7379* nor other mutations on the chromosome contribute to BBB breakdown, and indicating that *pasiflora1* is cell-autonomously required.

In the vicinity of the *pasiflora2* locus, no P-element insertions were available. Since the gene belongs to the same family, we decided to pursue *pasiflora2* using RNAi. Moreover, the KK line is very potent as it causes strong BBB permeability (Fig. 1D,F) and embryonic lethality with *repo-Gal4* (1% hatch). The fact that an impaired BBB is observed in the glial-specific knockdown suggests that *pasiflora2* is also cell-autonomously required. To exclude off-target effects, we tested two additional RNAi lines that target different sequences of the mRNA. With pan-glial expression, the VDRC line GD43952 and an shRNAi line that we generated (TRiP design; Ni et al., 2011) show qualitatively similar defects of dye penetration and embryonic lethality, with milder defects observed in the GD line (Fig. 1D; data not shown). For all our experiments with *pasiflora2*, we used the KK RNAi line.

To exclude the possibility that the leaky BBB is a result of earlier defects in glia specification and/or migration, we analyzed the number and positions of SPG (Ito et al., 1995; Beckervordersandforth et al., 2008). We detect the full set of SPG in both *pasiflora1^Δ^* and *repoGal4;UAS-pasiflora2-RNAi* embryos, with somewhat variable positions of nuclei in both control and mutant embryos (Fig. 1G). In summary, our results show that *pasiflora1* and *pasiflora2* are novel genes with a specific role in BBB formation.

### Pasiflora genes are required for tracheal tube size and barrier function

**Fig. 2.**
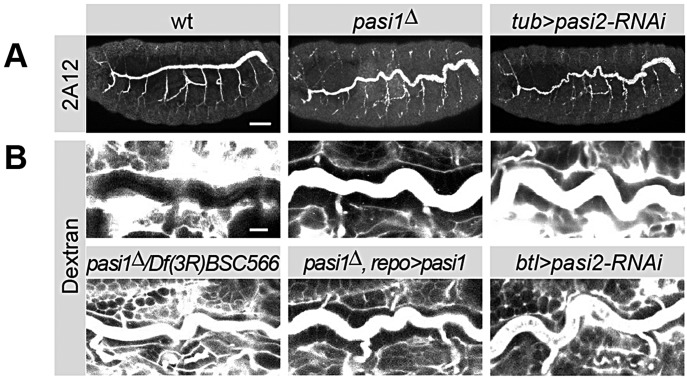
**Pasiflora genes are required for tracheal barrier formation and control of tube length.** (A) Lateral views of stage 16 embryos stained with 2A12. The dorsal trunks appear overelongated and convoluted in *pasiflora1* and *pasiflora2* mutants. Maximum projections of 16-18 µm *z*-stacks. *n*=8-10. (B) Single confocal sections of 20 h AEL dye-injected embryos of different genotypes. Dye-labeled dextran does not diffuse into the tracheal lumen of wt, but penetrates in *pasiflora1* and *pasiflora2* mutants. Glial overexpression of *pasiflora1* does not rescue the tracheal phenotype of *pasiflora1^Δ^*. Lateral views of dorsal trunk. *n*=5-16. Anterior is left and dorsal is up. Scale bars: 40 µm in A; 10 µm in B.

We noticed that the tracheal tubes of *pasiflora1^Δ^* do not fill with air (data not shown), indicating that the tracheal barrier is also compromised. To confirm this observation, we performed the dye penetration assay and visualized the dorsal trunks. In wt, the dye is excluded from the tracheal lumen, but it rapidly diffuses into the tubes of *pasiflora1^Δ^* homozygous, transheterozygous *pasiflora1^Δ^* over the two deficiency chromosomes and in embryos with ubiquitous knockdown of *pasiflora2* (*tubulin-Gal4*) (Fig. 2B; data not shown). Both mutants also show excessively elongated and convoluted dorsal trunks, a result that was confirmed by staining stage 16 embryos with the 2A12 antibody that recognizes the luminal protein Gasp (Fig. 2A). Overelongated dorsal trunks are observed in the majority of SJ mutants and are believed to be due, at least in part, to the role of SJs in the transcytosis of chitin deacetylases that terminate tube elongation (Luschnig et al., 2006; Wang et al., 2006; Dong et al., 2014). Knocking down *pasiflora2* with the more trachea-specific *breathless-Gal4* leads to qualitatively similar phenotypes but with lower penetrance (Fig. 2B). As expected, the tracheal defects are not restored in our rescue experiment with the glial driver, further supporting that *pasiflora1* is cell-autonomously required (Fig. 2B). Thus, *pasiflora1* and *pasiflora2* are required for tracheal barrier function and tube size control.

### Pasiflora genes are expressed in SJ-forming embryonic epithelia

**Fig. 3.**
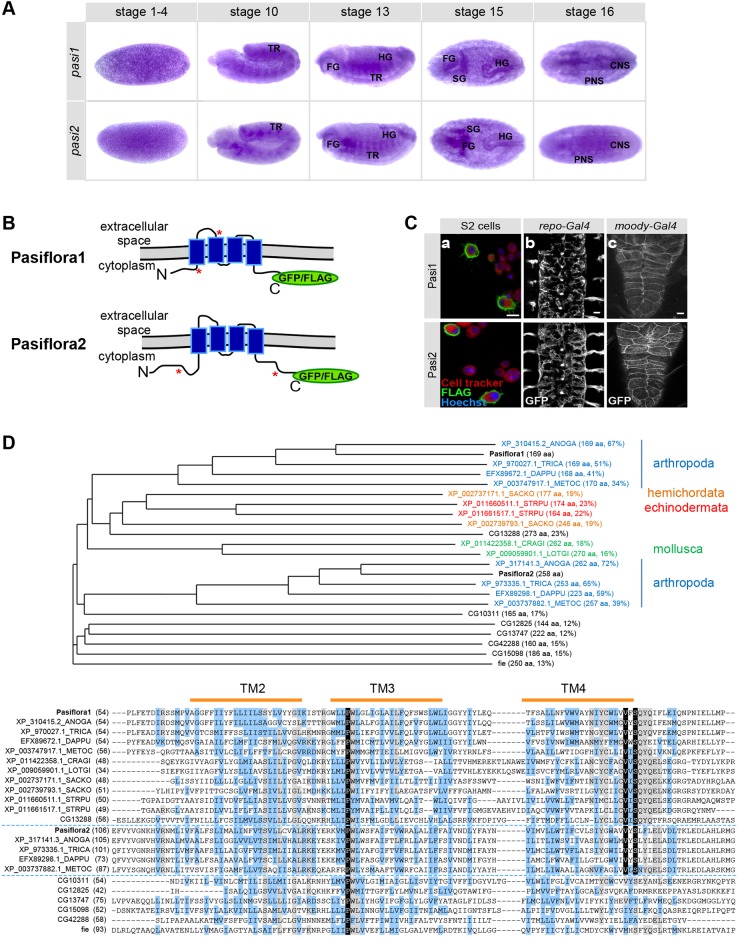
**Pasiflora1 and Pasiflora2 are conserved tetraspan membrane proteins co-expressed in embryonic epithelia.** (A) *In situ* hybridization with antisense probes for *pasiflora1* (*pasi1*) and *pasiflora2* (*pasi2*) in *w^1118^* embryos. Both genes are expressed maternally (stage 1-4). Zygotic transcripts are detected from stage 10 onwards in epithelia and nervous system. TR, trachea; FG, foregut; HG, hindgut; SG, salivary glands; CNS and PNS, central and peripheral nervous system. Anterior is left. (B) Predicted structure of pasiflora proteins. The site of fusion of GFP/FLAG is depicted by green ovals. The epitopes used for antibody production are highlighted with red asterisks. (C) Tagged pasiflora proteins localize at the plasma membrane. (a) Single confocal sections of S2 cells transiently transfected with Pasiflora1-FLAG or Pasiflora2-FLAG. (b) Ventral views of fixed stage 16 embryos expressing Pasiflora1-GFP or Pasiflora2-GFP in glia. Maximum projections of 7 µm *z*-stacks. (c) Third instar larval CNS expressing live-imaging Pasiflora1-GFP or Pasiflora2-GFP in SPG. Maximum projections of 10 µm *z*-stacks. Anterior is up. (D) Multiple sequence alignment and phylogenetic tree of pasiflora proteins and homologs. Shown is a section of the alignment centered on TM domains 2-4, with start positions as indicated; identical residues are highlighted in black, strongly similar residues in blue, residues conserved in a majority of proteins in gray. The length of each protein and the degree of sequence identity/similarity to Pasiflora1 or, in the case of Pasiflora2 orthologs, to Pasiflora2, are indicated in parentheses in phylogenetic tree labels. For full protein sequences, see the supplementary Materials and Methods. Scale bars: 10 µm in Ca,b; 20 µm in Cc.

To characterize the expression pattern of *pasiflora1* and *pasiflora2*, we performed RNA *in situ* hybridization in wt embryos. The genes show identical expression patterns throughout embryogenesis (Fig. 3A). Ubiquitous weak expression is first detected at stages 1-4, suggestive of maternal contribution. Zygotic transcripts are detected in epithelial tissues from stage 10 onwards. The tracheal placodes are labeled at stage 10 and the anterior hindgut at stage 11; expression persists in these tissues throughout development. During stages 14-16, trachea, foregut, hindgut, epidermis and salivary glands are marked. At stage 16, we detect weak staining in the nervous system and labeling of some cells that, based on their position, are likely to be exit and/or peripheral glia. A clearer *in situ* for *pasiflora1* showing similar expression is displayed on the BDGP website (http://insitu.fruitfly.org). Therefore, both genes are specifically expressed in embryonic epithelia and insulating glia – all tissues that form SJs.

Several attempts to generate specific antibodies recognizing the two proteins were unsuccessful. Overexpression of the highly hydrophobic pasiflora proteins was toxic to the bacteria. We therefore raised antibodies against a mixture of two peptides (Fig. 3B; see supplementary Materials and Methods for epitopes), but unfortunately neither sera nor affinity-purified antibodies showed specific labeling in embryos (data not shown).

### Molecular features of the pasiflora family

Pasiflora1 and Pasiflora2 are small proteins of 169 and 258 amino acids, respectively, with four TM domains but no signal peptide. Their predicted topology is very similar, with intracellular N- and C-termini and a larger first extracellular loop (Fig. 3B). To examine whether the proteins localize at the plasma membrane or some intracellular membrane compartment, we analyzed their subcellular localization *in vivo* and in cell culture. We tagged both proteins with GFP and FLAG, attached to an alanine-rich linker (Fig. 3B), and expressed them in Schneider cells (S2) as well as glia, which we imaged in stage 16 embryos (*repo-Gal4*) and third instar CNS (*moody-Gal4*). We find that pasiflora proteins localize at the plasma membrane *in vivo* and in S2 cells (Fig. 3C). Importantly, C-terminal tagging with GFP does not seem to affect protein function, as pan-glial expression of *pasiflora1-GFP* rescues the leaky BBB of *pasiflora1^Δ^* at a level similar to that of untagged *pasiflora1* (Fig. 1E,F).

Although the protein topology of pasiflora proteins resembles that of claudins, they show no sequence similarity to this or other tetraspan families. However, Pasiflora1 orthologs are readily identified in all arthropods, including insects, arachnidae and crustacea, as well as in molluscs, echinoderms and hemichordates, suggesting that the protein predates the protostome-deuterostome divide. Alignment of the best protein matches from species within these different phyla indicates conservation along the entire length of the protein, with two absolutely conserved motifs: a PW motif at the beginning of TM3 and a VxSQYQ motif that straddles the boundary between TM4 and the C-terminal intracellular domain. Pasiflora2 orthologs are found within arthropods but not beyond; they share the PW motif in TM3 and a shortened VxS motif at the boundary of TM4. Interestingly, the sequence comparison shows that Pasiflora1 is more closely related to its orthologs in other phyla than to Pasiflora2, with a sequence similarity of 15%, suggesting that the separation of the two family members is ancient (Fig. 3D). Consistent with this significant sequence divergence, we find that pasiflora proteins act non-redundantly, since pan-glial expression of *pasiflora1*, but not *pasiflora2*, rescues the BBB phenotype of *pasiflora1^Δ^* (Fig. 1E; supplementary material Fig. S1).

Pasiflora proteins were previously shown to belong to a family of otherwise uncharacterized tetraspan proteins with similar length and topology in *Drosophila* and *Anopheles* (With et al., 2003)*.* Based on our current analysis, we find seven additional members in this wider family (CG13288, CG13747, CG15098, CG12825, CG10311, CG42288 and Fire exit); however, with the exception of CG13288, which closely resembles Pasiflora1 (23% identity), these proteins are slightly more diverged and only share the PW motif in TM3 (Fig. 3D). Interestingly, the original founding member *Fire exit*, which is the most strongly diverged, is expressed in exit and peripheral glia but no molecular or biological function has been demonstrated (With et al., 2003). Whereas pan-glial knockdown of *Fire exit* causes adult subviability (19% survivors; 51% for negative control), knockdown of the other family members did not impair viability (*CG10311*, *CG12825*, *CG13747*, *CG15098*) or was not performed owing to a lack of RNAi strains in the collection (*CG13288*, *CG42288*).

Different lines of evidence indicate that *pasiflora1* and *pasiflora2* are co-expressed. First, RNA *in situ* hybridization showed that the genes are similarly expressed in embryonic epithelia (Fig. 3A). Second, both genes were identified as differentially expressed in embryonic glia based on microarray transcriptome profiling (U.G., unpublished). Third, based on developmental RNA-seq, the genes are part of co-expression clusters with SJ genes (*kune*, *cold*; *sinu*, *Nrx-IV*, *Mcr*, *Gli*, *crok*, *cold*, *crim*) (Graveley et al., 2011). The notion that *pasiflora1* and *pasiflora2* expression is tightly co-regulated is also supported by the observation that both genes, together with more than half of the known SJ component-encoding mRNAs, are predicted targets of miR-184 (Hong et al., 2009; Iovino et al., 2009).

Based on the phenotypic analysis, the expression patterns and the targeting by miR-184, we hypothesized that pasiflora proteins are either SJ components themselves or play a role in complex assembly and/or trafficking. However, neither of the proteins was found in an MS-based proteomic analysis of Mega complexes that succeeded in identifying at least ten known SJ components, possibly because of their small size (Jaspers et al., 2012). Notably, the claudins Sinu and Kune, which are of similar size and structure to the pasiflora proteins, were also not detected in the MS analysis.

### Pasiflora genes are required for the localization of SJs

**Fig. 4.**
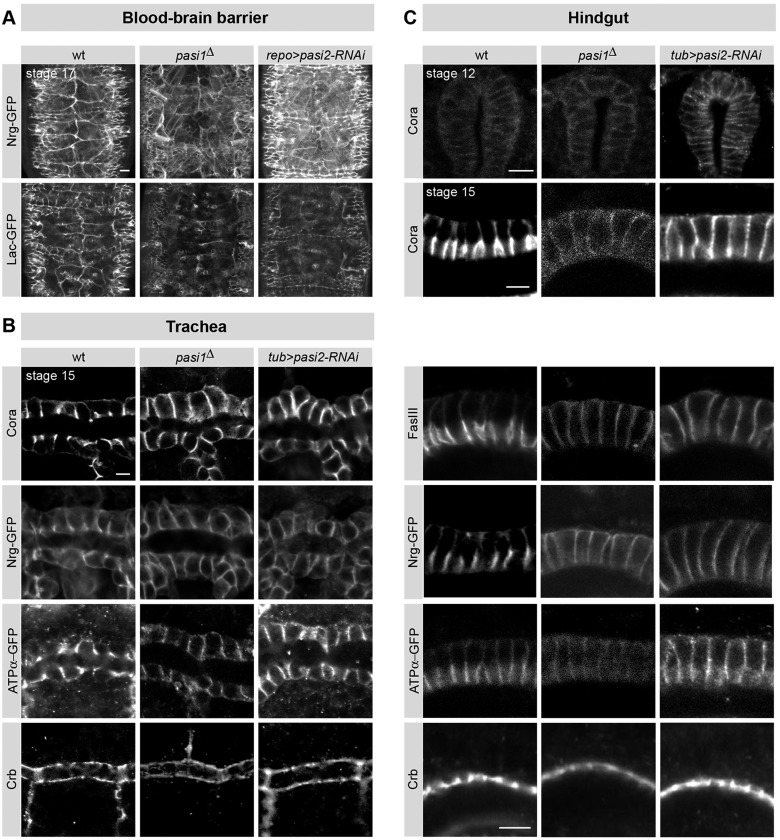
**Pasiflora genes are specifically required for localization of SJs.** (A) Ventral surface views of nerve cord of 20 h AEL embryos expressing the live-imaging SJ markers Nrg-GFP and Lac-GFP. SPG SJs are severely disrupted in pasiflora mutants. Maximum projections of 8-11 µm *z*-stacks. Anterior is up. *n*=5-16. (B) Single confocal sections of stage 15 dorsal trunks stained for different junctional proteins. In pasiflora mutants, SJ proteins spread basolaterally. Cell polarity is preserved, as revealed by Crb staining. *n*=6-12. (C) Single confocal sections of stage 12 and 15 hindguts stained for SJ proteins and Crb. In pasiflora mutants, SJ proteins localize at the lateral membrane, similar to wt at stage 12, but fail to restrict apicolaterally at stage 15. Crb localization is preserved. *n*=5-21. Scale bars: 10 µm in A and in C stage 12; 5 µm in B and in C stage 15.

To confirm that *pasiflora1* and *pasiflora2* play a role in SJ development, we analyzed the morphology and subcellular localization of SJs in the mutants. We first visualized the embryonic BBB using the endogenously expressed live-imaging markers Nrg-GFP and Lac-GFP (Morin et al., 2001). In wt late stage 17 embryos, both markers label SJs and trace the outlines of SPG, which make continuous contacts with their neighbors to seal the CNS. In *pasiflora1^Δ^* and *repo-Gal4;UAS-pasiflora2-RNAi* embryos, SJs appear discontinuous and severely disorganized (Fig. 4A), demonstrating that both genes are required for SJ formation in the embryonic BBB.

SPG are very large but thin cells, complicating the visualization of SJ localization along the lateral membrane during embryonic stages. We therefore examined the hindgut and tracheal columnar epithelia. In the hindgut of wt stage 12 embryos, SJ proteins accumulate evenly along the lateral membrane, but at stage 15 are restricted to the apicolateral membrane compartment, as revealed by staining for Cora. In *pasiflora1^Δ^* and *tubulin-Gal4;UAS-pasiflora2-RNAi* embryos, Cora localizes similarly to wt at stage 12, but fails to restrict apicolaterally at stage 15 and remains distributed along the lateral membrane. At stage 15 we observe similar mislocalization of additional SJ markers, such as Nrg-GFP, ATPα-GFP and FasIII (Fig. 4C). Notably, in the variably penetrant phenotype of *pasiflora2-RNAi*, the mislocalization phenotype is more pronounced for Cora and FasIII followed by Nrg, whereas ATPα is only mildly mislocalized. SJs are also mislocalized in tracheal cells. In the tracheal epithelium of wt stage 15 embryos, Cora, ATPα-GFP and Nrg-GFP accumulate in the apicolateral membrane, but spread basolaterally in the mutants (Fig. 4B). Furthermore, the intensity of SJ proteins appears somewhat lower in the mutants. Dimmer SJ immunostaining has been reported for other SJ mutants as well, but in the cases where it was tested by western blots, the protein levels were, overall, not reduced (Genova and Fehon, 2003; Paul et al., 2003). This suggests that the weaker staining is not a result of reduced transcription or increased protein degradation, but rather a consequence of the dispersed localization. In addition, *pasiflora2* mutants show milder defects compared with loss of *pasiflora1*, which is likely to be due to incomplete knockdown of *pasiflora2* with RNAi as compared with genomic knockout of *pasiflora1* in the deletion mutant. In addition to *w^1118^*, we also tested genetically closer controls; i.e. a viable line with precise excision of *P{EP}G4182* and embryos with ubiquitous knockdown of a random RNAi (VDRC KK 108356) and confirmed that they have wt localization of SJs (data not shown). Collectively, our results show that *pasiflora1* and *pasiflora2* are required for the apicolateral localization of SJs in hindgut and tracheal epithelia.

SJs also play an earlier, independent role in maintaining cell polarity by restricting the size of the apical Crb domain (Laprise et al., 2009). To investigate if cell polarity is disturbed in pasiflora mutants, we analyzed the distribution of Crb in hindgut and trachea. In both tissues, Crb localizes at the apical membrane similarly to wt (Fig. 4B,C). Thus, in columnar embryonic epithelia, *pasiflora1* and *pasiflora2* selectively affect SJ organization but not the establishment or maintenance of cell polarity.

### Pasiflora proteins localize at the SJ and their localization depends on other complex components

**Fig. 5.**
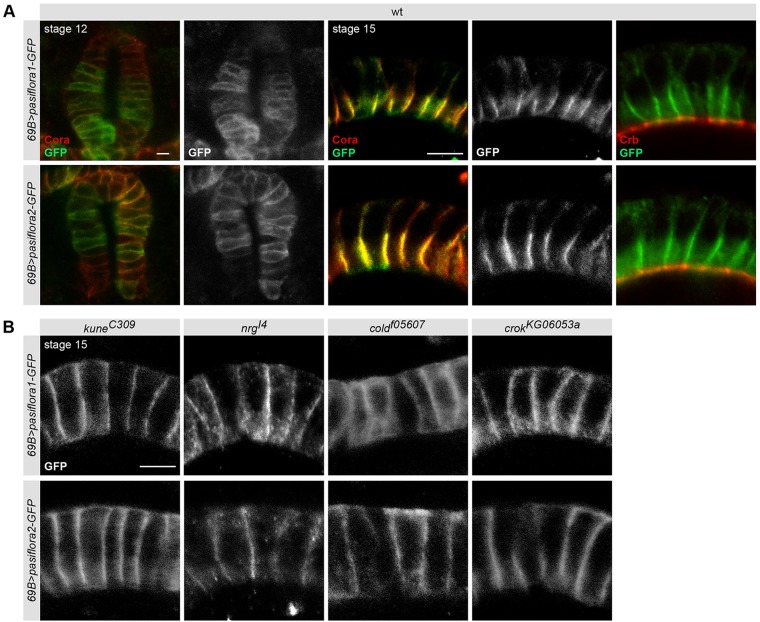
**Pasiflora proteins localize at SJs dependent on other complex components.** Single confocal sections of hindguts of fixed embryos expressing Pasiflora1-GFP and Pasiflora2-GFP. (A) In wt, both proteins colocalize with Cora at SJs, but not with Crb. (B) In embryos mutant for different SJ genes, Pasiflora1-GFP and Pasiflora2-GFP lose their apicolateral accumulation and spread basolaterally. *n*=5-11. Scale bars: 5 µm.

To determine if pasiflora proteins accumulate in a specific membrane compartment, we analyzed the localization of GFP-tagged versions in the hindgut epithelium using *69B-Gal4*. Both Pasiflora1 and Pasiflora2 colocalize at the membrane with Cora, but not Crb, and display the characteristic dynamic expression of SJ proteins: at stage 12, they localize along the lateral membrane and at stage 15 become restricted apicolaterally (Fig. 5A). Occasionally, we detect a small amount of pasiflora proteins, but not Cora, at the apical and basolateral membranes. We believe that this is due to overly high protein levels under Gal4-UAS expression because we only observe it in a minority of cells and it correlates with the strength of expression in the given cell. Altogether, our results show that Pasiflora1 and Pasiflora2 are membrane proteins localizing at SJs.

Core SJ proteins are known to be interdependent for localization, and removal of one component is sufficient to destabilize the entire complex and mislocalize other SJ proteins. To address whether Pasiflora1 and Pasiflora2 localization is similarly affected, we analyzed their distribution in the hindgut of stage 15 embryos homozygous for amorphic mutations in SJ proteins. Co-staining for Cora served as a readout of SJ integrity. In *kune^C309^* and *Nrg^I4^* core component mutants, as well as in *cold^f05607^* and *crok^KG06053a^* embryos, both pasiflora proteins and Cora lose their restricted localization and extend basolaterally (Fig. 5B; data not shown). In summary, our results show that pasiflora proteins localize at the SJ and their localization depends on other SJ proteins, suggesting that they are core complex components.

### Pasiflora and core SJ proteins show interdependent mobility within the membrane

To further show that pasiflora proteins are core SJ components, we performed a series of FRAP experiments. In the epidermis of wt stage 15 embryos, when mature SJ complexes are established, the fluorescence of GFP-tagged core SJ proteins exhibits slow recovery after photobleaching because the stable SJ complexes are very large and move slowly within the membrane. In mutants of core components or of proteins involved in complex assembly, the SJ complex is not properly formed and the free GFP-linked proteins can diffuse rapidly to the bleached region (Oshima and Fehon, 2011).

**Fig. 6.**
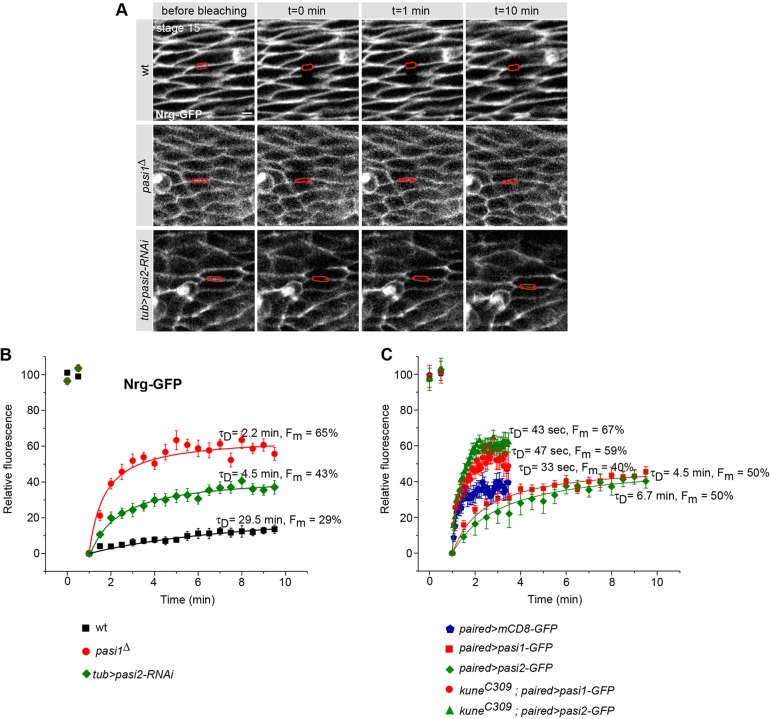
**Pasiflora proteins are core SJ components.** (A,B) *pasiflora1* and *pasiflora2* are required for SJ complex formation. (A) Single confocal sections of lateral epidermis of stage 15 embryos expressing live-imaging marker Nrg-GFP. After photobleaching, Nrg-GFP diffuses slowly in the wt, but rapidly in pasiflora mutants. Bleached membranes are marked in red. (B) Quantification of relative fluorescence of Nrg-GFP over time in different genotypes. (C) Quantification of relative fluorescence of Pasiflora1-GFP and Pasiflora2-GFP over time. In wt, pasiflora proteins are less mobile than mCD8-GFP, and in cells with disrupted SJs (*kune* mutant) they diffuse rapidly into the bleached region. *n*=9-17. Error bars indicate s.e.m. Scale bar: 5 µm.

To determine if SJ complex formation is impaired in pasiflora mutants, we performed FRAP of Nrg-GFP in the epidermis of stage 15 embryos. For our analysis, we extracted from the fitting procedure the percentages of mobile fractions (*F*_m_) and, more importantly, the characteristic time of diffusion (τ_D_) [see supplementary Materials and Methods for half time (t_1/2_) and detailed analysis]. In wt, Nrg-GFP shows very slow recovery and even 10 min after bleaching only 10% of the fluorescence has recovered. Recovery has not reached a plateau, but the strong embryo movements did not allow us to systematically perform longer time-lapse recordings. For Nrg-GFP in wt we extrapolate τ_D_=29.5 min and 29% mobile fraction. By contrast, in both *pasiflora1^Δ^* and *tubulin-Gal4;UAS-pasiflora2-RNAi* embryos, Nrg-GFP recovers rapidly (τ_D_=2.2 and 4.5 min, respectively) and has a large mobile fraction (65% and 43%, respectively) (Fig. 6A,B). Notably, fluorescence never recovers to 100% in our experiments or those of others (Laval et al., 2008; Oshima and Fehon, 2011), but the nature of this immobile fraction is currently unclear. Therefore, the behavior of Nrg-GFP in pasiflora mutants is similar to that observed in mutants of SJ core components and proteins involved in complex assembly. Together with their localization at SJs, these results argue that pasiflora proteins are core components required for the formation of SJ complexes.

**Fig. 7.**
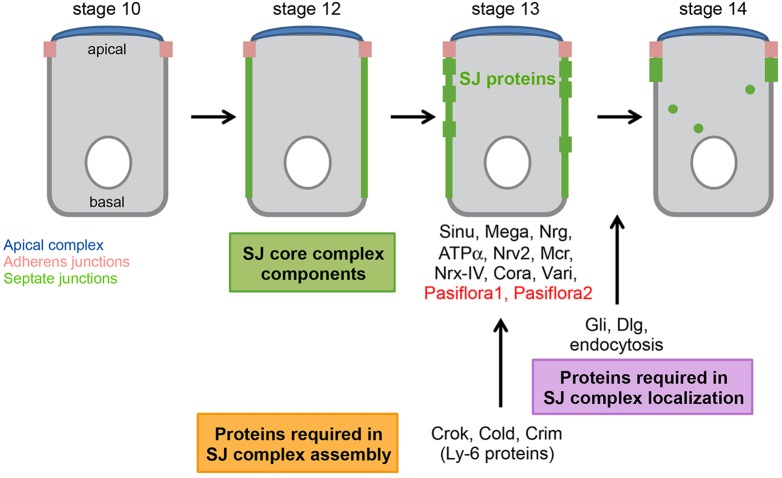
**Timeline and players in SJ morphogenesis.** The SJ complex consists of several core components, including the novel pasiflora proteins. Ly-6 proteins are required for the assembly of (sub)complexes at stage 13, while endocytosis and the SJ proteins Gli and Dlg are essential for complex relocalization at stage 14. Modified from Oshima and Fehon (2011).

To further show that pasiflora proteins are integral SJ components, we analyzed their mobility within the membrane in the epidermis of stage 15 embryos. We used *paired-Gal4* and expressed GFP-tagged pasiflora proteins in epidermal stripes. As a control, we used membrane-tagged mCD8-GFP and imaged embryos at stage 14, at a time when SJs are not yet mature and diffusion within the plasma membrane should not be impeded. mCD8-GFP recovers remarkably quickly (τ_D_=33 s) and its mobile fraction is 40%. By contrast, the recovery of Pasiflora1-GFP and Pasiflora2-GFP is significantly slower (τ_D_=4.5 and 6.7 min, respectively), with their mobile fractions being 50% (Fig. 6C). The faster recovery of Pasiflora1-GFP and Pasiflora2-GFP proteins compared with Nrg-GFP could be due to the overexpression conditions. Therefore, pasiflora proteins are more immobile than other TM proteins, suggesting that they are part of a membrane complex. To ultimately show that this is the SJ complex, we analyzed the mobility of Pasiflora1-GFP and Pasiflora2-GFP proteins in epidermal cells of *kune^C309^* mutants, which have disrupted SJs, and observed that both proteins lose their restricted mobility and diffuse very rapidly (Pasiflora1-GFP, τ_D_=47 s, *F*_m_=59%; Pasiflora2-GFP, τ_D_=43 s, *F*_m_=67%) (Fig. 6C). Taken together, these results validate that pasiflora proteins are indeed core SJ components (Fig. 7).

## DISCUSSION

### Pasiflora1 and Pasiflora2 are novel SJ core components

We have identified two previously uncharacterized proteins, Pasiflora1 and Pasiflora2, as novel components of the *Drosophila* SJ. Several lines of evidence support this notion. First, *pasiflora1* and *pasiflora2* mutants exhibit all the characteristic phenotypes associated with disrupted SJs: breakdown of blood-brain and tracheal barriers, overelongated dorsal trunks, and SJ mislocalization in a variety of tissues. In the BBB, SJs appear severely disorganized and in columnar epithelia SJ proteins fail to localize at the apicolateral membrane and instead spread basolaterally. Second, the genes are co-expressed in embryonic epithelia that rely on SJs for their function and the proteins overlap with Cora at the apicolateral membrane. Similar to known SJ proteins, pasiflora localization depends on other complex members, as they spread basolaterally in SJ mutant backgrounds. Finally, using FRAP we demonstrate that pasiflora proteins are core SJ components. In stage 15 epidermal cells, Nrg-GFP displays limited lateral mobility after photobleaching owing to its incorporation in the large multi-protein complex. By contrast, in pasiflora mutants, Nrg-GFP diffuses rapidly, indicating that SJ complex formation is compromised. Overexpressed pasiflora proteins also move slowly within the membrane of wt cells, but diffuse rapidly in cells with disrupted SJs, showing that they are themselves associated with the SJ complex.

An emerging idea is that not all SJ proteins are as interdependent as previously thought and that distinct subcomplexes exist within the large, highly ordered, multi-protein complex. Our observations and those of others (Nelson et al., 2010; Oshima and Fehon, 2011; Hall et al., 2014) indicating that in SJ mutants the localization of other complex members is differentially affected and that the fluorescence of GFP-tagged SJ proteins does not fully recover after photobleaching support this notion.

### Potential roles of the pasiflora family

Pasiflora proteins are conserved in arthropods and beyond and share the global topological features of the tetraspan superfamily, with short conserved sequence motifs. The ability of different tetraspan families to form ribbons based on homo- and heterotypic interactions in *cis* within the plasma membrane suggests that pasifloras, together with claudins, are involved in forming the highly regularly spaced septa of the SJ. Freeze-fracture experiments have shown that SJs form ribbons, with an apparent size of a single septum of 10 nm and a regular spacing of 15-20 nm. Depending on the tissue, these ribbons are either highly aligned with each other (mature ectoderm) or meandering (developing wing disc) (Fristrom, 1982; Lane and Swales, 1982; Furuse and Tsukita, 2006). In the SJ, the plasma membranes of neighboring cells are not fused but closely juxtaposed at a distance of 15 nm and there is no evidence in invertebrates that different tissues have distinct paracellular permeability. Claudins and pasifloras are therefore unlikely to create pores in *trans* with specific size and charge selectivity. This suggests that the small claudins and pasifloras act only in *cis* to form ribbons, while the single-pass membrane proteins of the complex mediate the *trans* interaction with the neighboring cell via their large extracellular adhesive domains. To date, the structural basis for the intermolecular interaction between tetraspan proteins has not been resolved (Krause et al., 2015). The pasiflora proteins belong to a larger family with nine members in *Drosophila*. We have shown that Pasiflora1 and Pasiflora2 are expressed in embryonic epithelia and glia and act non-redundantly during SJ formation. Little is known about the other family members: *Fire exit* is expressed in exit and peripheral glia, which also form SJs; *CG15098* is expressed in the midgut, which forms structurally different, smooth SJs.

Our study reveals that the composition of the SJ complex strongly resembles that of other junctional and TM protein complexes, where adhesive or signaling receptors are embedded in a complex environment of hydrophobic tetraspan proteins of different types, in this case three different claudins and two different members of the novel pasiflora family. Membrane complexes such as the SJ are particularly refractory to biochemical and structural analysis owing to their hydrophobicity and large size. However, due to their crucial function in all invertebrates and the vertebrate paranode, it is possible, by genetic means, to identify and study the structural core components as well as the biogenesis of the complex. Given the medical importance of the paranodal SJ in particular and of tetraspan proteins in general, our discovery of pasiflora proteins opens the possibility of studying these proteins and their interactions in a highly accessible and sensitive paradigm.

## MATERIALS AND METHODS

### Fly strains and constructs

For generation of transgenic lines we used the ɸC31 integrase method and inserted constructs in attP2 and attP40 docking sites (Groth et al., 2004; Markstein et al., 2008; Pfeiffer et al., 2010). Rescue constructs were generated by PCR amplification from cDNA clones RE54605 (*pasiflora1*) and LD42595 (*pasiflora2*) [*Drosophila* Genomics Resource Center (DGRC), Indiana, USA]. Tagged proteins were generated by fusion of *Drosophila*-optimized GFP (pJFRC14, Addgene) or 3×-FLAG to the C-terminus after an alanine-rich linker. For *in vivo* expression, pJFRC2 (*10x-UAS*) was used (Addgene). For S2 cell expression, the pMT vector was used (metallothionein promoter). The *pasiflora2*-shRNAi line was generated according to Ni et al. (2011) by inserting a 21 nt hairpin (sense strand: TACAATGTGATTATGGTGCTC) in pWalium20 [Transgenic RNAi Project (TRiP), Harvard Medical School, Boston, USA]. *pasiflora1^Δ^* was generated by imprecise excision of *P{EP}G4182* [Bloomington *Drosophila* Stock Center (BDSC)]; the deletion spans the region 17794826-17796435. For fly strains obtained from published sources see the supplementary Materials and Methods. For live genotyping, *Kruppel-Gal4;UAS-GFP* or *Dfd-YFP* balancers were used (BDSC). All strains were raised at 25°C.

### Embryonic dye penetration and viability assay

The dye permeability assay was performed as previously described (Schwabe et al., 2005). CNS dye penetration was quantified using a custom Definiens (http://www.definiens.com) script that automatically measures pixel intensity after excluding overexposed areas. Mean pixel intensity was taken as readout value. To assess significance, one-way ANOVA was performed over all groups with Student-Newman-Keuls post-hoc test.

To measure lethality, stage 15 embryos were dechorionated, rinsed, mounted on a coverslip coated with heptane glue, covered with halocarbon oil (Huile 10S VOLTALEF), and placed on an agar plate facing a pile of yeast. Embryos were followed during late embryogenesis and larval life and the stage at which they died was scored.

### Immunohistochemistry, live imaging and RNA *in situ* hybridization

Immunohistochemistry of embryos was performed following standard procedures. For antibodies used see the supplementary Materials and Methods. Live imaging of embryos was performed as described (Schwabe et al., 2005). Dissected third instar CNS was mounted in PBS and imaged directly. S2 cells were transfected with *pMT-Pasiflora-FLAG*, induced with 0.2-0.5 mM CuSO_4_ 24 h post-transfection, and fixed 24 h post-induction. All confocal images were acquired using an LSM 710 system and ZEN acquisition software (Carl Zeiss). Image analysis was performed using ImageJ (NIH).

Whole-mount *in situ* hybridization on embryos was performed as previously described (Lehmann and Tautz, 1994) with the following modifications: the post-fix step between embryo rehydration and proteinase K treatment was removed, and incubation with anti-DIG antibodies was overnight at 4°C. Antisense probes were generated by *in vitro* transcription from RE54605 (*pasiflora1*) and LD42595 (*pasiflora2*).

### FRAP experiments and analysis

Embryos were dechorionated, rinsed, mounted on coverslips with glue, and covered with halocarbon oil. Imaging and photobleaching were performed with a c-Apochromat 40×/1.20 W Korr M27 objective. Two images were acquired before photobleaching and GFP was bleached using maximal output power of a 488 nm laser. The bleached membrane was located in the lateral epidermis and was approximately 3 μm in length. A time series of images was started immediately after photobleaching, with one image every 30 s for 10 min, except for *paired-Gal4;UAS-mCD8-GFP* and *kune^C309^;paired-Gal4,UAS-pasiflora1/2-GFP* for which images were captured every 4 s for 3 min. A home-written Definiens script was used for correction of embryo movements and a second script for extraction and normalization of fluorescence intensity of photobleached membranes at each time point. Data were fitted to an equation for one-dimensional free diffusion; characteristic time of diffusion and percentages of mobile fractions were then extracted. For detailed analysis of FRAP data see the supplementary Materials and Methods.

### Alignment and phylogenetic analysis

PSI-BLAST and manual inspection were performed to identify orthologs and *Drosophila* paralogs of pasiflora proteins (for a list, see the supplementary Materials and Methods). Protein sequences were aligned and a phylogenetic tree was constructed using the ClustalW algorithm as implemented in Vector NTI 11.5 (Life Technologies). Protein topologies were verified using SMART-EMBL.

## Supplementary Material

Supplementary Material
