# Supplementary material for: Pasiflora proteins are novel core components of the septate junction
Source: Development. 2015 Sep 1;142(17):3046–57. doi: 10.1242/dev.119412 (PMC4582180; doi:10.1242/dev.119412)
Supplement: Supplementary Material [file supp_142_17_3046__index.html]

Supplementary Material 

# Pasiflora proteins are novel core components of the septate junction

## DEV119412 Supplementary Material

- Supplementary Material
