# Supplementary material for: Pasiflora proteins are novel core components of the septate junction
Source: Development. 2015 Sep 1;142(17):3046–57. doi: 10.1242/dev.119412 (PMC4582180; doi:10.1242/dev.119412)
Supplement: Supplementary Material [file supp_142.17.3046_DEV119412supp.pdf]

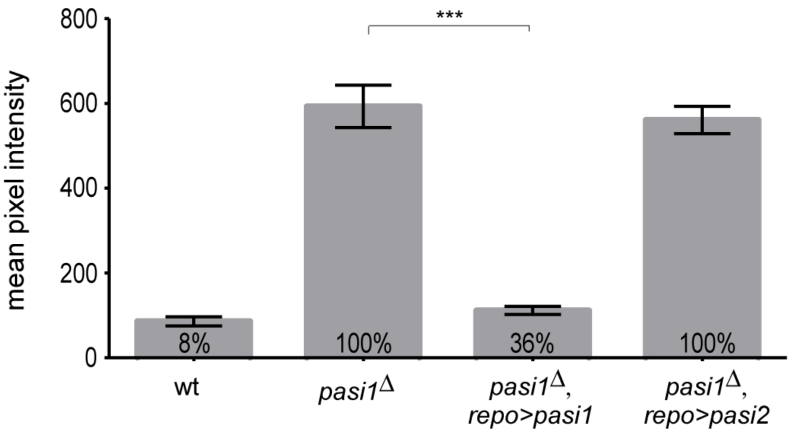


**Supplementary Fig. S1. *pasiflora* genes act non-redundantly during BBB formation.** Quantification of the dye penetration assay in 20 h AEL dye-injected embryos. Pan-glial over-expression of *pasiflora1*, but not *pasiflora2* rescues the dye penetration of *pasiflora1*$^{\Delta}$. Columns represent intensity of dye penetration into the nerve cord as measured by mean pixel intensity. The percentage of embryos showing penetration is indicated at the bottom of each column. Brackets and asterisks indicate significance of pairwise comparisons. *** $p<0.001$, ±SEM, n=20-45.

## Supplementary Materials and Methods

### Fly strains

The following fly strains were obtained from published sources: *repo-Gal4* (V. Auld), *moody-Gal4* (Schwabe et al., 2005), *tubulin-Gal4* (E. Arama), GFP traps $Nrg^{G00305}$, $Lac^{G00044}$, and $ATP\alpha^{G00109}$ (W. Chia), *UAS-mCD8-GFP* (L. Luo), *UAS-dicer2* and *UAS-RNAi* lines $pasiflora1^{KK102223}$, $pasiflora2^{KK105806}$, $pasiflora2^{GD43952}$, and $lkb1^{KK108356}$ (Vienna *Drosophila* Research Center, VDRC, Austria), *Df(3R)BSC566*, *Df(3R)ED5785*, $nrx\text{-}IV^{4304}$, $nrg^{I4}$, $kune^{C309}$, $cold^{f05607}$, $crok^{KG06053a}$, *69B-Gal4*, *paired-Gal4* and *breathless-Gal4* (Bloomington *Drosophila* Stock Center, BDSC, Indiana, USA). $w^{1118}$ was used as wt.

### Immunohistochemistry

Primary antibodies used were: Developmental Studies Hybridoma Bank, DSHB antibodies mouse anti-Repo (8D12), mouse IgM anti-Gasp (2A12), mouse anti-Cora (C615.16), mouse anti-FasIII (7G10), and mouse anti-Crb (Cq4) (all used 1:5), rabbit anti-GFP (A11122, Molecular

Probes, 1:100). Alexa- (Molecular Probes) and Cy- (Jackson ImmunoResearch) conjugated secondary antibodies were used at dilutions of 1:400 and 1:200, respectively.

**Production of antibodies**

For each protein two 15-16 amino acids-long peptides were synthetically generated and their mixture was injected in rabbit and guinea pig (for Pasiflora1) and hen (for Pasiflora2) (Eurogentec, Seraing, Belgium). The epitopes were: for Pasiflora1: `SPLFETDIRSSMPVA`, `IIWSDNVRTGSYAVA`, and for Pasiflora2: `NLHSKMSRSTRSVRI`, `STANSLAGSRPTTPHS`. The sera, as well as affinity-purified antibodies were tested by immunostainings in wt embryos in various concentrations (including 1:2).

**Analysis of FRAP data**

*Image Registration and analysis*. Embryo movements are unavoidable and pose severe challenges for the analysis of time-lapse recordings. We used a home-written *Definiens* script to correct for lateral drift and non-linear distortions of the raw confocal images due to changes of cellular shape. In brief, for a confocal stack of n images with index 1..n, a *built-in* image registration algorithm was first applied to three reference images with rounded indexes n/6, n/2 and 5n/6, respectively (the middle image was used as reference image for registration). The remaining images were then registered with respect to the reference image closest in index number. Given the strong embryo movements and drift that we observed, this strategy ensured a more robust alignment compared to a registration procedure based on only one reference image for the whole stack.

A second *Definiens* script was then used to automatically extract the fluorescence intensity trajectories of the photobleached membrane regions. To detect the photobleached region we applied to registered images a 2D-Gaussian filter with a kernel size of 5x5x3 pixels, followed by an edge 3D filter. This filter is sensitive to signal variations between successive time-lapse images, and is thus ideal to detect the photobleached region that exhibits a strong decrease in fluorescence intensity just after the photobleaching step. The average fluorescence intensity in the identified region can then be extracted for each time point, and normalized with

respect to its maximal and minimal values at the time points before and immediately after the photobleaching step, respectively.

*FRAP data analysis.* In a first approximation, the diffusion in the thin photobleached membrane can be modelled by one-dimensional free diffusion. The experimental data were fitted to the empirical formula given in equation (1), which agrees within 5% with the solution of the diffusion equation in one dimension for recovery into an interval of zero intensity (Ellenberg et al., 1997; Ellenberg and Lippincott-Schwartz, 1999)

$$I(t) = I(final)\left(1 - \sqrt{\frac{\tau_D}{\tau_D + \pi \cdot (t - t_0)}}\right) \qquad \mathbf{(1)}$$

with I(t) = intensity as a function of time; $t_0$= time right after photobleaching; I(final) = final intensity reached after complete recovery; $\tau_D$ = characteristic time of diffusion.

The fitting procedure was performed using *Origin 8.5*. We kept $t_0$ constant, and extracted I(final) and $\tau_D$ from the fitted curves. Mobile fractions were calculated as ratios of fluorescence intensity in the bleached area after recovery of the signal to fluorescence intensity before photobleaching.

Another common approach used to analyze FRAP recovery curves is the calculation of half-time ($t_{1/2}$) as the time required for the bleached fluorescence to recover to half of its maximum recovery value (Yguerabide et al., 1982; Oshima and Fehon, 2011). We extracted $t_{1/2}$ from exponential fits of the recovery curves and found for Nrg-GFP $t_{1/2}$= 0.7±0.1 minutes, 1.1±0.1 minutes and 7.6±5.4 minutes in *pasiflora1*$^{\Delta}$, *tubulin-Gal4;UAS-pasiflora2-RNAi* and wt embryos, respectively. For overexpressed (*paired-Gal4*) Pasiflora1-GFP and Pasiflora2-GFP, we calculated $t_{1/2}$= 0.9±0.1 minutes and 1.9±0.2 minutes, respectively, while for the control membrane-bound mCD8-GFP $t_{1/2}$=11±1 seconds. For overexpressed Pasiflora1-GFP and Pasiflora2-GFP in the *kune*$^{C309}$ mutant background, we calculated $t_{1/2}$=18±2 and 16±0.7 seconds, respectively. All these values are in the same order of magnitude with both the characteristic times of diffusions $\tau_D$ calculated using a one-dimensional free diffusion model and the results

obtained by Oshima and Fehon., 2011. Minor quantitative differences between our results and those of Oshima and Fehon (e.g. mobile fractions of mCD8-GFP) might result from the usage of different drivers (*paired-Gal4* vs *engrailed-Gal4*) and pipelines of data analysis.

Furthermore, a puzzling observation is that our recovery curves exhibit I(final) values in the range of 40-60%, whereas values close to 100% would be expected from full recovery of diffusing GFP-tagged proteins. This reveals the presence of an immobile or extremely slow fraction of diffusing GFP-tagged proteins within our observation time. The presence of such an immobile SJ fraction has also been observed before (Laval et al., 2008; Oshima and Fehon, 2011), but its nature remains largely obscure. One of the factors contributing to the two distinct populations of diffusing proteins may lie in the genetics. *pasiflora1* is studied in zygotic mutant embryos although the gene is also maternally expressed and *pasiflora2* by RNAi; these incomplete loss of function conditions contribute to the broader distribution of fluorescence recovery times. However, more surprisingly, partial recovery is also observed for the membrane-bound mCD8-GFP, suggesting the existence of additional sources of traps for the proteins. One possibility is that at early stages, before fully stable insulating SJ complexes have formed, SJs already operate as a fence limiting free mobility within the plane of the membrane, thus hindering diffusion of a fraction of the GFP-tagged proteins.

**Supplementary references**

**Ellenberg, J., and Lippincott-Schwartz, J.** (1999). Dynamics and mobility of nuclear envelope proteins in interphase and mitotic cells revealed by green fluorescent protein chimeras. Methods **19**, 362-372.

**Ellenberg, J., Siggia, E.D., Moreira, J.E., Smith, C.L., Presley, J.F., Worman, H.J., and Lippincott-Schwartz, J.** (1997). Nuclear membrane dynamics and reassembly in living cells: targeting of an inner nuclear membrane protein in interphase and mitosis. The Journal of cell biology **138**, 1193-1206.

**Laval, M., Bel, C., and Faivre-Sarrailh, C.** (2008). The lateral mobility of cell adhesion molecules is highly restricted at septate junctions in Drosophila. BMC cell biology **9**, 38.

**Oshima, K., and Fehon, R.G.** (2011). Analysis of protein dynamics within the septate junction reveals a highly stable core protein complex that does not include the basolateral polarity protein Discs large. Journal of cell science **124**, 2861-2871.

**Yguerabide, J., Schmidt, J.A., and Yguerabide, E.E.** (1982). Lateral mobility in membranes as detected by fluorescence recovery after photobleaching. Biophysical journal **40**, 69-75.

## Protein sequences of Pasiflora orthologs and paralogs

```
>gi|23170782|gb|AAN13416.1| CG8121, isoform C [Drosophila melanogaster]
MMNYGRKTPSTYRSNPSVYSHATGRSSTNLHSKMSRSTRSVRIPWYQRPLLKNNQYIDIQKGAMLVGLFA
IFLSLFTIATSIFDIYCYAMAAPGSTHYGYYIISYEFVYVGNKHVRNMLIVFALFSLIMALINFVTSVLL
CVALRKEYERKVMPWLWSFAIFTVWRALALIFFAIVNDLYFAYNVIMVLLWTIFCVLSIYGWAVVYSLFL
ELVDLTKLEDLAHLRMGTMASLHASTANSLAGSRPTTPHSTVSTMPVG

>gi|7300307|gb|AAF55468.1| CG7713, isoform A [Drosophila melanogaster]
MVVLSSCWSPIIWSDNVRTGSYAVAGYTAALSAVMITLISYMLAGGESAQLYSPLFETDIRSSMPVAGGF
FIIYFLLIILSSYLVYYGIKISTRGWLLPWLGLIGLAILFQFSWSLWLIGGYYIYLEQTFSALLNFVWVA
YNIYCWLVVFSQYQIFLEIQNPNIELLMP

>gi|7300137|gb|AAF55304.1| CG10311, isoform A [Drosophila melanogaster]
MPKPLLNSCCLCQSTRNGSVISGILAIVLSIITIVVIFTTRVHFKTIIFDFIPNDIVKIILVINLCMTIL
ISLLMIIGALKRNHYLMVPWVVLGIMIAIGLLISVIYTGIVFFIDGYVLTGVLWLIFGLIVCAIMTYCWC
VVYSEYANLSEENERGRYNKQPYRR

>gi|7304157|gb|AAF59194.1| CG12825 [Drosophila melanogaster]
MWKLDTKGGIICSGCLSIAFAITYLVLMDDYFWKYGLYEMGIHISALQILGSVVLIVGAIKQKHKFFVPW
MITTGFFLYLMVNLFISLIVQGTAWIFGPLMVVPFTAYLGCALYSVQKAFDRMRKEEPPAYASLSDKKEF
INHI

>gi|7292397|gb|AAF47802.1| fire exit, isoform A [Drosophila melanogaster]
MLLPTVSGQSAAGCKWKNCCRRSWEMLMAPVSPKNLRTTALLTSIYQLLISHCALFLVLLGLAHAEQMCE
VLELDILDQKDNGFYNMSPFHNDLRLQTAAQLAVATENLLYVMAGIAGTYALSAIFLFFGVFKNRPGLII
PWLVVEFLLMIGLGALVFMLRDTKIVQLLGGQVPYFIICYILICMDYCKWYVMHSFYQSLRTMNKLREIA
TVAIPCPAPGAIPYRFQREHMYLGSNGYKHILTESPDGQC

>gi|7303961|gb|AAF59004.1| CG13747 [Drosophila melanogaster]
MAPNDPSHPGGKSTAVAMATSHTISYVQCQNLKYNVIILGWLGVIISTVILGSSALTIHFRPDIELLLNQ
WPMNLVPVAEQQLLINLLTIFSSIVYGLSLINMGVSLLLLIGIARDSSCLIYPWLIYHGVIFGFGLYLGV
FYATAGLFIDLSSFLMCLLVFNLVLVIFYKIYHEVFTLFRVMEQLSKDGGMGGLYYQDAEHGWTAAGVPF
QHVYVPRLPMQK

>gi|45445476|gb|AAF57628.2| CG15098 [Drosophila melanogaster]
MLSRVFCMRLNTYGVVIGWLGVILSFLATILLSLALGFVDEIAQQIAKGSEDSNKTATEIRSVLIIVFSV
YLALKVINLLASAMLVAGTVKERHLLLLPWLINNGVLLVFGIITNIAMLAQIIGNTSFLSALPIIFVDVG
LLLLTWYLYYGIYSLFKQIQASREIQRPLIPQPTQQTNSYPSYTKI
```

>gi|442630457|ref|NP_001261455.1| CG13288, isoform C [Drosophila melanogaster]
MAILESCCFWKDVRSGSFACAIYTLVYFGFSTLMFLFYLIEEQDFLLGNRAQPLGESLLEKGDVTVVTVI
FNILLLFCSILMVLSSVLLILGLQQNKRHLLIPWISFMLGDLLIEVCHLVHLALSRRVKFDPIVGFIFTM
DFFLLCLNLYCLLCVISQYQTFRSQRAEMRLAASTASPIVVFTAAEKLTKAQQQQLHNNLLQQLETGSGY
GKRRRMLGAASPLITSQRPTNFSTITEEEEQQSRTDHLTEQPTGEDLGRIPIFTLASSPAHCH

>gi|220902215|gb|AAF58297.3| CG42288, isoform A [Drosophila melanogaster]
MACRCKYLKQIFTKCCYCYSLRFGVLLFGCIFLTWFIYITIGTGFMMECIFPNEYQRSLIPAPAALKATM
VFSFFGIIVSAMLCLGVHNNNEMLFLPFLVFTPIWIIVHIFALTVYSFNTIIIILTVITMLLLVYAWLVV
WSYYIELLFAYEDELETGYV

>gi|58380261|ref|XP_310415.2| AGAP003856-PA [Anopheles gambiae str. PEST]
MVWLESCWSPFIWTNSVKSGSYAVAAYTVATSIVLITMTTYNLLGGDSSQLYSPLFETDIRDSMQVVGGF
FILYFILLILSAGGVCYSLKTTTRGWMLPWLALFGIAILFQFVFGLWLIGGYYIYLQQTLSSLILWVWMA
YNIYCWMCVYSQYQIFENMQSPNIELLMP

>gi|91076134|ref|XP_970027.1| PREDICTED: uncharacterized protein LOC658556 [Tribolium castaneum]
MVILTSCWSPCIWTNDLKTSCKVIAFYTVAMSIVVMTFIVFDMSGGDSTQLYNPLFEADIRYSMQVVGTC
SMIFFSSLIISTILLVVGLHKMNRGMMLPWLIEFGIVVLFQLVFGLWLLGGYYIYLETVLHTFVIWSWMT
YNGYCWLVVYSQYQVFEEMQSPNIELLWP

>gi|321478715|gb|EFX89672.1| hypothetical protein DAPPUDRAFT_230163 [Daphnia pulex]
MPSISTCWSPCFWHMNVREGTFAVAFYTTFFPIVIIAYTAYVMRGGDSAQFYLPYFEADVKDTMQSVGAI
AILFCLMFIICSFMLVQGVRKEYRGLFFPWMICMTLVVLFQAVYGLWIIIGYYILWNVFVSIVNWIWMAA
NMYFFMCVWSQYEIVTELQARHIELLYP

>gi|391347330|ref|XP_003747917.1| PREDICTED: uncharacterized protein LOC100898088 [Metaseiulus occidentalis]
MPILDRCWNPLMMYHFTNVRFGSYYCAVYTMVLHIVFIFYAIYAIQGGRTEWFFSPYFEYSQRGTTAAAS
ITIVLCLIFLFFTFLLCRGVRRDNRCMYFPWMMGMTIEILLMIGVGLWYIVRYYRNLFSVLAAILLWTID
GFHIYCLLIVISQYQIVKNLQEPKFEFLYP

>gi|291239765|ref|XP_002739793.1| PREDICTED: uncharacterized protein LOC100371028 [Saccoglossus kowalevskii]
MYDMAIIVRCCCCNVRTGSIWAASYSLITSLLNVGLYAYFTVIYQNMESGYLHIPYIVFPITTGCSGLVF
MLSIVLIIGLIMDHKKLLLPWIIFYIFYILAEGATSFVLVFFFGAHAMNLFALAWFVSRTILGVYCIICV
VSQYQELNEGRGRSYDYERDAYMDDDADAPPETIESPRVMKFTIHGDQTQRQYSTASTKSAGDIELEETR
SYSTPRRHPRSPKIVPREVQTPNNGYQNRYFEDDVF

>gi|291234470|ref|XP_002737171.1| PREDICTED: lysosomal-associated transmembrane protein 4B-like [Saccoglossus kowalevskii]
MTIITTCCCCMNTRTGTIVSGIYTTILAGIRLIMMLSALASAGDKAGGLISSYIIDLLLLIGLLIVSIIL
IYGAIKDNRILLVPWMVFMVIFIILTLITTIIIIVYASFSSWVTWSNIIWFVIFNTINIYCLLCVISQYQ
ELKEGRGTADYHRQLAASRGRPVVVTTTTTPARVEKA

>gi|780177238|ref|XP_011661517.1| PREDICTED: uncharacterized protein LOC100891165 isoform X2 [Strongylocentrotus purpuratus]
MAILTRCCCFDNVRTGSKACGVYSLIYCLVSVGILLWEFVDYIEMSSYTGAARKGLYAAYSIDLVVLIFL
FIASIILLVGVAKNSKCMLIPYMVAIPLLIVLQLISWIIVFVFIGMGEVAEHVIDIAQCVVWFVVTAPKI
LCLICVLSQYQELKDGRGRQYNMV

>gi|780170733|ref|XP_011660511.1| PREDICTED: uncharacterized protein LOC105436567 [Strongylocentrotus purpuratus]
MAIMQSCCCFSNVRSGSLACGVFSLITSCFYLAEGSWNLALAVSVHSNNTGPAIDGTYAAYSIDIIVDVF

LLIASIVLLVGVSVNNRTMLIPYMVAIMAVMVLQAITWLIQIVFIGAAYIVLIVILVVWLLYTAFNIMCL
LCVISQYQELSEGRGRAMQAQWSTPITERSTLVR

>gi|762168016|ref|XP_011422358.1| PREDICTED: uncharacterized protein LOC105324851 [Crassostrea gigas]
MAIVKTFLCFSNTRVGTLICGCYSLVNSLICLVFYLFRYVGYQLIEDSQEYKGIVYAGFVLYGLMIAASL
ILIPGVQLDKRYLLLPWVYVLILNVLYETGSIALLTTVHMEREKTLNAWEIVWMFYYCFKLIANCYCFAC
VVSQYQELSEGRGTYDYLYKPRNRRSMGRAREFDIDTFELPFGVHLPPYAESDPCKELNPPLYENLYPQP
KLEDRRSIDIRQIGLCHNVQTVNQHSERYNYSQRYSGDMPDRYSITVDVVWI

>gi|676473692|ref|XP_009059901.1| hypothetical protein LOTGIDRAFT_165135 [Lottia gigantea]
MTDWFTSDLIKVISVVCLGFYLFRYIALDAISKSIEFKGIIYAGFVLYSLLIAVSLILLPGVYMDKRYLM
LPWIYVLVVTVLYETGAVALLTTVHLEYEKKLQSWEIISVCFYMLRLVANCYCFACVVSQYQELTEGRGT
YEFLYKPSSSSPSDVEVYTPPFGANLPPYSIEDQHKEFSPPPYDNLSFETESDDVFRTATSACIHGECRH
DLLNDISECCAPDVTSRVDRYDPEYGVLQDAMSGSVLTHPVPEYRLPGIDYQMTTHLTWL

>gi|118789020|ref|XP_317141.3| AGAP008322-PA [Anopheles gambiae str. PEST]
MNYGRKTPSTYRSTPSVYSHITSRSQSNLHSQRSRSMKSVMVPWYQKPLLHNNKYLDIQRGAFIMGLFSI
FVAIFTIGTAVFDIYCLALAAPGSSHYGYYIISYEFVYVGNVHVRNALMVAALFSLIGGLVVLVTSIMLV
HALRKEYESKIVPWLWSFAIFTLFRLLAFLFFSIVNDLIFAYNILITLIWSVIIVASLYGWLMVYSLYLE
LADLTKLEDLAHLRMGTMQSLNASVAHSLAGSRPTTPHSTVSTMPVGDTKFI

>gi|91085253|ref|XP_973335.1| PREDICTED: uncharacterized protein LOC662125 [Tribolium castaneum]
MNYGKKSPTTGTPSVYSHVTTRSSANLRSSRSIKSLKIPWYRKPIITESYILDVQRSSLLIGVFSLLLSI
FTVITSCFDLYCYSMAVPGSVHTGYYVISYQFIYVGNRHVRNTLVMFAAFSILLAIGVFVTSIMLIIALR
KEYEKKMVPWLYAFGIFTVFRLLAYLFFSIVNDMIFAYNVLMCLLWTVFLAISIYGWILVYSLYIELSDL
TRLEDLAHLRMGTMQSLNASTVPSLAGSRPTTPHSTVSTMPVG

>gi|321478341|gb|EFX89298.1| hypothetical protein DAPPUDRAFT_126746 [Daphnia pulex]
MSRSMKSLRVAWYKKPILQDAFFTDIQTGSMITAIFSMVISLFTIATAVLDIYCLGMTKPGVTHYGYYIM
SFQFVYVGNGNVRNTLIVFALFSAIAAVALFVTSCILLKALRKEIEKEMVPWLFASGVFFVWRTIAIIFA
SVVNDMIFGYHIAMCLFWVAFILLGTLGWVIVYSLYLELSDLTKLEDLAHLRMGTMSSLNMTQSIAGSRP
TTPHSTVSTAQVV

>gi|391326765|ref|XP_003737882.1| PREDICTED: uncharacterized protein LOC100900661 [Metaseiulus occidentalis]
MGSRSVLNKTTSTIDSSRYRKRQRWYQRRPFLDNAYFTNLQKGSYIAAIYALIECLFMICLVIFDIYCLA
EAQPGSRHFRYFGISFLFVYSGNQHVRNLLITVSVISFIGAMFLLVTSAQIISALRKEQEARFRPWLYSM
AAFTVWRFCAIIFRSIANDLYYAYHILMLLIWAALIAGNVFAGLVVLSNYQELSDITRLEDLARSKMGGS
TSVSRSVSHNSLDTFKNSSVHNSSFQSNYSNRSGVPSVSSVPMRQLR
